# Utility of Candidate Genes From an Algorithm Designed to Predict Genetic Risk for Opioid Use Disorder

**DOI:** 10.1001/jamanetworkopen.2024.53913

**Published:** 2025-01-09

**Authors:** Christal N. Davis, Zeal Jinwala, Alexander S. Hatoum, Sylvanus Toikumo, Arpana Agrawal, Christopher T. Rentsch, Howard J. Edenberg, James W. Baurley, Emily E. Hartwell, Richard C. Crist, Joshua C. Gray, Amy C. Justice, Joel Gelernter, Rachel L. Kember, Henry R. Kranzler

**Affiliations:** 1 Mental Illness Research, Education and Clinical Center, Crescenz Veterans Affairs Medical Center, Philadelphia, Pennsylvania; 2 Center for Studies of Addiction, Department of Psychiatry, University of Pennsylvania Perelman School of Medicine, Philadelphia; 3 Department of Psychological and Brain Sciences, Washington University School of Medicine, St Louis, Missouri; 4 Department of Psychiatry, Washington University, St Louis, Missouri; 5 Veterans Affairs Connecticut Healthcare System, West Haven; 6 Department of Internal Medicine, Yale School of Medicine, New Haven, Connecticut; 7 Faculty of Epidemiology and Population Health, London School of Hygiene & Tropical Medicine, London, United Kingdom; 8 Department of Biochemistry and Molecular Biology, Indiana University School of Medicine, Indianapolis; 9 BioRealm LLC, Walnut, California; 10 Department of Medical and Clinical Psychology, Uniformed Services University, Bethesda, Maryland; 11 Department of Psychiatry, Yale University School of Medicine, New Haven, Connecticut; 12 Departments of Genetics and Neuroscience, Yale University School of Medicine, New Haven, Connecticut

## Abstract

**Importance:**

Recently, the US Food and Drug Administration gave premarketing approval to an algorithm based on its purported ability to identify individuals at genetic risk for opioid use disorder (OUD). However, the clinical utility of the candidate genetic variants included in the algorithm has not been independently demonstrated.

**Objective:**

To assess the utility of 15 genetic variants from an algorithm intended to predict OUD risk.

**Design, Setting, and Participants:**

This case-control study examined the association of 15 candidate genetic variants with risk of OUD using electronic health record data from December 20, 1992, to September 30, 2022. Electronic health record data, including pharmacy records, were accrued from participants in the Million Veteran Program across the US with opioid exposure (n = 452 664). Cases with OUD were identified using *International Classification of Diseases, Ninth Revision*, or *International Classification of Diseases, Tenth Revision*, diagnostic codes, and controls were individuals with no OUD diagnosis.

**Exposures:**

Number of risk alleles present across 15 candidate genetic variants.

**Main Outcome and Measures:**

Performance of 15 genetic variants for identifying OUD risk assessed via logistic regression and machine learning models.

**Results:**

A total of 452 664 individuals with opioid exposure (including 33 669 with OUD) had a mean (SD) age of 61.15 (13.37) years, and 90.46% were male; the sample was ancestrally diverse (with individuals of genetically inferred European, African, and admixed American ancestries). Using Nagelkerke *R*^2^, collectively, the 15 candidate genes accounted for 0.40% of variation in OUD risk. In comparison, age and sex alone accounted for 3.27% of the variation. The ensemble machine learning. The ensemble machine learning model using the 15 variants as predictive factors correctly classified 52.83% (95% CI, 52.07%-53.59%) of individuals in an independent testing sample.

**Conclusions and Relevance:**

Results of this study suggest that the candidate genetic variants included in the approved algorithm do not meet reasonable standards of efficacy in identifying OUD risk. Given the algorithm’s limited predictive accuracy, its use in clinical care would lead to high rates of both false-positive and false-negative findings. More clinically useful models are needed to identify individuals at risk of developing OUD.

## Key Points

**Question:**

How well do genetic variants from an opioid use disorder (OUD) risk algorithm, recently given premarketing approval by the US Food and Drug Administration, perform in a large, independent sample?

**Findings:**

In a case-control study of 452 664 individuals, the 15 genetic variants collectively accounted for 0.40% of the variation in OUD risk. In an independent sample, a machine learning model composed of the 15 variants correctly identified case and control status 52.83% of the time.

**Meaning:**

These results suggest that the genetic variants included in the algorithm do not meet reasonable standards of efficacy in identifying OUD risk and that more clinically useful models are needed to identify individuals at risk of developing OUD.

## Teaser

This case-control study assesses whether 15 genetic variants from an algorithm purported to predict risk for opioid use disorder (OUD) were associated with OUD risk in a population of US veterans.

## Introduction

Opioid misuse and opioid use disorder (OUD) are significant public health problems. In 2022, 6.1 million US residents 12 years or older met criteria for OUD,^1^ with 94.8% acknowledging misuse of prescription analgesics and 40.9% reporting receipt of the misused medication from a physician.^1^ Given a surge in opioid overdose deaths,^2,3,4^ efforts have been made to identify individuals at risk of opioid misuse.

Common genetic variation accounts for a small proportion of differences in OUD liability.^5^ Polygenic scores derived from common genetic risk variants (ie, single nucleotide variants [SNVs]) across the genome account for less variance in OUD risk (3.74%) than sociodemographic factors (41.32%).^6^ Nevertheless, attempts have been made to develop and commercialize genetic risk algorithms for OUD.^7,8,9^ These models usually include a few SNVs in genes considered causal candidates based on their presumed effect on neural reward systems. In addition to these candidate variants having small effects,^10^ few have been substantiated in genome-wide association studies, a more rigorous method of identifying risk variants.^11,12,13^

Genetic predictive models are also vulnerable to confounding based on differences in patterns of genetic similarity.^14^ If the prevalence of OUD varies among individuals with historically different geographic origins (either due to real differences or biases in the data used to train a machine learning [ML] model), the model will falsely attribute predictive power to SNVs that are markers of genetic similarity rather than disorder risk. Such spurious associations arise from population stratification, differences in allele frequencies that result from historical migration and mating patterns (eFigure 1 in Supplement 1). These models are likely to bias predictions and lead to the false conclusion that they are useful for predicting risk of complex traits like OUD.

The US Food and Drug Administration (FDA) recently gave premarketing approval to an algorithm (AvertD) that incorporates 15 SNVs to predict OUD risk.^15^ The package insert for the algorithm states that the “… 15 detected genetic polymorphisms are involved in the brain reward pathways that are associated with OUD…,” but it provides no citations to support the associations, all of which appear to have been identified through candidate gene studies.^16^ The algorithm was developed among 1381 US individuals and tested in a multicenter clinical study that enrolled 812 patients, of whom 385 were selected by a statistician for inclusion in the analyses. The manufacturer reports sensitivity of 82.76% and specificity of 79.23%.^16^ In this case-control study of a sample of 452 664 US veterans, we assessed (1) whether the SNVs were individually associated with OUD, (2) how much variance in OUD risk the SNVs accounted for collectively, (3) whether the SNVs were associated with genetic similarity rather than OUD risk, and (4) whether basic demographic characteristics (ie, age and sex) more accurately estimated OUD risk than the SNVs.

## Methods

### Participants

The Million Veteran Program (MVP), an initiative of the US Department of Veterans Affairs, was approved by the Central Veterans Affairs Institutional Review Board (IRB) and all site-specific IRBs. All participants provided written informed consent, and this study followed the Strengthening the Reporting of Observational Studies in Epidemiology (STROBE) reporting guideline for case-control studies.

Using electronic health record data, we identified genotyped patients enrolled in MVP who had filled an outpatient opioid analgesic prescription. Cases were defined based on having at least 1 *International Classification of Diseases*, *Ninth Revision*, or *International Classification of Diseases*, *Tenth Revision*, OUD code. Controls were opioid-exposed individuals with neither an OUD diagnosis code nor any prescription fill for medications commonly used to treat OUD. As sensitivity analyses, we removed cases who had received only 1 outpatient OUD diagnosis code and reran all models. The sample’s genetically inferred ancestry (GIA) composition was assigned based on patterns of similarity to reference genomes of individuals in the 1000 Genomes project.^17^ Categories, or groups, included were European, African, admixed American, East Asian, South Asian, and unassigned (Table 1, eFigure 2 in Supplement 1).

**Table 1.** Opioid Use Disorder Case-Control Status Across GIA Groups

GIA superpopulation^b^	Any opioid exposure		Short-term opioid exposure^a^	
	No. of cases	No. of controls	No. of cases	No. of controls
All	33 669	418 995	3704	121 810
European	20 392	284 986	1982	82 859
African	9496	85 118	1211	22 910
Admixed American	3215	39790	411	12 586

Abbreviation: GIA, genetically inferred ancestry.

^a^ Indicates 4 to 30 days.

^b^ Based on genetic similarity to global superpopulations defined by the 1000 Genomes Project.

Among opioid-exposed individuals, we also selected a subset with only short-term documented exposure to opioids (4-30 days total), as this reflects the approach used by the manufacturer to evaluate its algorithm.^9^

### Genotyping and Imputation

MVP samples were genotyped using a custom array for MVP, release 4 (Affymetrix Axiom Biobank Array; Thermo Fisher Scientific). Quality control and imputation were performed by the MVP Genomics Working Group.^18^ Duplicate samples and those with a sex mismatch, 7 or more relatives in MVP (kinship >0.08), excessive heterozygosity, or a genotype call rate of less than 98.5% were removed. Monomorphic variants and variants with high missingness (call rate <0.8) or a Hardy-Weinberg equilibrium *P* < 1 × 10^−6^ were removed. Genotypes were phased with the SHAPEIT4 algorithm^19^ and imputed using Minimac software, version 4,^20^ with biallelic SNVs imputed using a hybrid of the 1000 Genomes Phase 3 and the African Genome Resources reference panel by the Sanger Institute.

To infer similarity to reference genomes and generate principal components to account for differences in genetic similarity, genetically inferred ancestry (GIA) composition was calculated by the MVP gwPheWAS (Genome-Wide × Phenome-Wide Association Study) Working Group.^21^ Further details on how subgroups were derived from genetic similarity principal components analysis (PCA) coordinates can be found in Hunter-Zinck et al.^18^ In summary, a random forest classifier was trained on the reference dataset using the first 10 PCs. The algorithm was applied to the MVP (PCA) data, and GIA was inferred when the classifier’s predicted probability was greater than 50%. Individuals with lower assignment probabilities were retained in the full-sample analyses. Although guidelines on the use of population descriptors in human genetics research^22^ were published after GIA analyses were conducted in MVP, we follow the recommendations as closely as possible.

### Statistical Analysis

#### Single Association Analyses of SNVs

We conducted association analyses for each of the 15 candidate SNVs with OUD case-control status using logistic regressions in PLINK, version 2.0.^23^ Analyses were also performed within GIA groups and the subset of individuals with short-term opioid exposure. To account for nonindependence, we randomly removed 1 individual from each pair of related individuals (n = 24 585). Three sets of analyses were conducted: (1) with no covariates, (2) including the first 10 genetic similarity PCs, and (3) including age, sex, and genetic similarity PCs as covariates.

#### Combined-SNV Regression Analyses

Using the glm() function in R, version 4.3.2 (R Project for Statistical Computing), we fit logistic regressions to examine the association of the SNVs with OUD status, including all 15 SNVs in a single regression model. We followed a procedure similar to that used with the single-SNV models, performing analyses in the full sample, the subsample with short-term opioid exposure, and GIA groups. We also ran 3 sets of models with increasing levels of adjustment, akin to the single-SNV models. We calculated Nagelkerke *R*^2^ to estimate the proportion of variance accounted for by each model and the area under the receiver operating characteristic curve (AUROC) to evaluate performance.

#### ML Models

We developed ensemble ML models in individuals with opioid exposure, those with short-term opioid exposure, and within GIA groups. To evaluate the ability of ML to identify OUD and its sensitivity to population stratification, we developed additional models in which case-control status was completely confounded by genetic ancestry. In these models, OUD cases comprised one GIA group and controls were randomly selected from another,^14^ creating an extreme example of how biases in the patterns of population stratification within training datasets can inflate model performance. These confounded models contrast (1) models that combine all ancestry groups and have moderate imbalances in population structure that reflect both natural variation in ancestry representation and disparities in OUD diagnosis due to social biases in health care settings and (2) within-ancestry models with balanced case-control distributions that account for differences in genetic similarity, effectively reducing bias. ML analyses were performed using the caret package in R, version 4.3.2 (R Project for Statistical Computing).^24^

Dosage data for each of the 15 SNVs were recoded as hard calls, reflecting the number of risk alleles for each individual. Of the 15 variants, 8 were directly genotyped and 7 were imputed, all with imputation quality greater than 0.8. In all models, controls were undersampled to yield equal proportions of cases and controls, which was required to address the severe imbalance in cases relative to controls. In the completely confounded models, we sought to preserve the greatest number of cases. Thus, all cases from the more common GIA group and a random sample of controls from the less common GIA group were included. We used a 75%-25% data split to obtain independent training and testing sets. We used 10-fold cross-validation during training, which systematically validated the model across subsets of the data. We used the default tuning parameter search feature of the caret package, with the optimal parameters selected based on the AUROC to provide balance between sensitivity and specificity.

We selected as base ML models 2 random forest implementations, rf (Breiman and Cutler random forests) and ranger (recursive portioning with random forests). We also used a linear support vector machine implementation (svmlinear2, using the e1071 library^25^ in the caret package) to approximate the method used for the FDA-approved algorithm, as the actual algorithm is proprietary.^9^ Base model–predicted probabilities were aggregated with the 15 SNVs to serve as predictive factors in a stacked ensemble ML model, which was trained using a binomial generalized linear model. This step allows the ensemble model to learn from the predictions of each of the base ML models to enhance its performance. Accuracy, sensitivity, and specificity were used to evaluate model performance. Accuracy, the proportion of all classifications that were correct, was calculated as (True Positives + True Negatives)/(True Positives + True Negatives + True Positives + False Negatives).

Model metrics were used to calculate a diagnostic odds ratio (DOR), indicating how much higher the odds were that the classifier produced a positive prediction in an individual with OUD than an individual without OUD. A 2-sided *P* < .05 indicated statistical significance.

## Results

Among 452 664 opioid-exposed individuals identified, including 33 669 cases with OUD, the mean (SD) age was 61.15 (13.37) years, 9.54% were female, and 90.46% were male. The sample’s GIA composition, assigned based on patterns of similarity to reference genomes of individuals in the 1000 Genomes project,^17^ was 67.46% European, 20.90% African, 9.50% admixed American, 0.81% East Asian, and 0.07% South Asian, with 1.25% unassigned (eFigure 2 in Supplement 1). In the subset of 125 514 individuals with documented short-term exposure to opioids, including 3704 individuals with OUD, mean (SD) age was 59.98 (14.84) years, 9.63% were female, and 90.37% were male. This subsample’s GIA composition was similar to that of the full sample (67.59% European, 19.22% African, 10.36% admixed American, 1.25% East Asian, 0.11% South Asian, and 1.47% unassigned).

In single-SNV models that did not account for genetic similarity, 13 of 15 SNVs were associated with OUD risk after Bonferroni correction (Table 2). Upon inclusion of measures of global genetic similarity, that number declined to 3 (Table 2). Five of the 10 SNVs no longer associated with OUD risk had opposite directions of effect in analyses that were uncontrolled. In analyses within GIA groups that accounted for local variations in genetic similarity, the 3 SNVs were associated with OUD risk only in individuals genetically similar to the European superpopulation. Similar results were obtained among individuals with short-term opioid exposure (eTables 1 and 2 in Supplement 2).

**Table 2.** SNV Associations With Opioid Use Disorder Case-Control Status Among Individuals With Opioid Exposure

SNV (gene)	Model type^b^	GIA superpopulation, OR (95% CI)^a^			
		African	Admixed American	European	All
rs7997012 (*HTR2A*)	Unadjusted	1.06 (1.00-1.12)	1.01 (0.95-1.06)	0.99 (0.97-1.01)	0.89 (0.88-0.91)^c^
	Adjusted	1.02 (0.96-1.08)	1.00 (0.95-1.06)	1.00 (0.98-1.02)	1.00 (0.98-1.02)
rs2236861 (*OPRD1*)	Unadjusted	1.03 (0.97-1.09)	1.02 (0.95-1.08)	1.04 (1.01-1.06)^d^	0.96 (0.94-0.98)^c^
	Adjusted	1.02 (0.96-1.07)	1.01 (0.95-1.08)	1.03 (1.01-1.06)^d^	1.03 (1.01-1.05)^d^
rs4680 (*COMT*)	Unadjusted	1.00 (0.96-1.03)	1.03 (0.98-1.09)	0.98 (0.96-1.00)	0.95 (0.94-0.97)^c^
	Adjusted	0.99 (0.95-1.02)	1.02 (0.97-1.07)	0.98 (0.96-1.00)^d^	1.01 (0.99-1.03)
rs1045642 (*ABCB1*)	Unadjusted	0.98 (0.94-1.02)	0.99 (0.94-1.05)	1.01 (0.99-1.03)	0.90 (0.89-0.92)^c^
	Adjusted	0.97 (0.93-1.00)	0.99 (0.94-1.04)	1.01 (0.99-1.03)	0.98 (0.97-1.00)
rs1800497 (*ANKK1*)	Unadjusted	0.97 (0.94-1.01)	0.99 (0.93-1.04)	1.06 (1.03-1.09)^c^	1.08 (1.06-1.10)^c^
	Adjusted	0.98 (0.94-1.01)	1.05 (0.99-1.11)	1.06 (1.03-1.08)^c^	1.03 (1.01-1.05)^c^
rs4532 (*DRD1*)	Unadjusted	1.04 (1.00-1.09)	0.99 (0.93-1.05)	1.02 (1.00-1.04)	0.93 (0.91-0.95)^c^
	Adjusted	1.03 (0.98-1.07)	0.97 (0.91-1.03)	1.02 (1.00-1.04)	1.01 (1.00-1.03)
rs948854 (*GAL*)	Unadjusted	0.99 (0.96-1.02)	1.00 (0.94-1.05)	1.02 (1.00-1.05)^d^	1.12 (1.11-1.14)^c^
	Adjusted	0.98 (0.94-1.01)	0.97 (0.91-1.03)	1.02 (1.00-1.04)	1.02 (1.00-1.03)
rs211014 (*GABRG2*)	Unadjusted	0.99 (0.95-1.02)	0.96 (0.90-1.03)	0.97 (0.94-0.99)^d^	1.04 (1.02-1.06)^c^
	Adjusted	0.99 (0.96-1.03)	0.96 (0.89-1.03)	0.97 (0.94-0.99)^d^	0.98 (0.96-0.99)^d^
rs1801133 (*MTHFR*)	Unadjusted	1.00 (0.96-1.06)	0.99 (0.94-1.04)	1.03 (1.00-1.05)^d^	0.94 (0.92-0.96)^c^
	Adjusted	0.99 (0.94-1.04)	1.04 (0.98-1.09)	1.02 (1.00-1.04)^d^	1.02 (1.00-1.04)
rs6347 (*SLC6A3*)	Unadjusted	1.00 (0.96-1.04)	1.04 (0.97-1.11)	1.00 (0.98-1.03)	1.11 (1.09-1.13)^c^
	Adjusted	1.01 (0.97-1.04)	0.99 (0.92-1.07)	1.00 (0.98-1.03)	1.01 (0.99-1.02)
rs1611115 (*DBH*)	Unadjusted	0.95 (0.91-1.00)^d^	0.92 (0.86-0.98)^d^	0.99 (0.97-1.02)	0.96 (0.94-0.98)^c^
	Adjusted	0.95 (0.90-1.00)^d^	0.96 (0.90-1.02)	0.99 (0.97-1.02)	0.98 (0.96-1.00)^d^
rs1051660 (*OPRK1*)	Unadjusted	0.97 (0.91-1.02)	1.06 (0.96-1.17)	0.98 (0.94-1.02)	0.98 (0.96-1.01)
	Adjusted	0.97 (0.91-1.02)	1.04 (0.94-1.14)	0.98 (0.94-1.01)	0.98 (0.95-1.01)
rs1799971 (*OPRM1*)	Unadjusted	1.03 (0.94-1.13)	0.88 (0.81-0.95)^c^	0.88 (0.85-0.92)^c^	0.82 (0.79-0.84)^c^
	Adjusted	0.99 (0.90-1.09)	0.91 (0.84-0.98)^d^	0.88 (0.85-0.91)^c^	0.89 (0.87-0.92)^c^
rs3758653 (*DRD4*)	Unadjusted	1.00 (0.95-1.04)	1.03 (0.97-1.09)	0.99 (0.96-1.02)	0.99 (0.97-1.02)
	Adjusted	1.00 (0.95-1.04)	1.07 (1.00-1.13)	0.98 (0.95-1.01)	1.00 (0.98-1.02)
rs9479757 (*OPRM1*)	Unadjusted	1.02 (0.97-1.06)	1.12 (1.02-1.21)^d^	1.07 (1.03-1.10)^c^	1.10 (1.07-1.12)^c^
	Adjusted	1.02 (0.98-1.07)	1.12 (1.02-1.21)^d^	1.07 (1.03-1.11)^c^	1.06 (1.03-1.08)^c^

Abbreviations: GIA, genetically inferred ancestry; OR, odds ratio; SNV, single nucleotide variant.

^a^ Based on genetic similarity to global superpopulations defined by the 1000 Genomes Project.

^b^ Unadjusted models include the 15 SNVs as predictive factors, while adjusted models also include the first 10 genetic similarity principal components as covariates.

^c^ Bonferroni-adjusted *P* = .003 (0.05/15).

^d^ *P* < .05.

**Figure 1.**
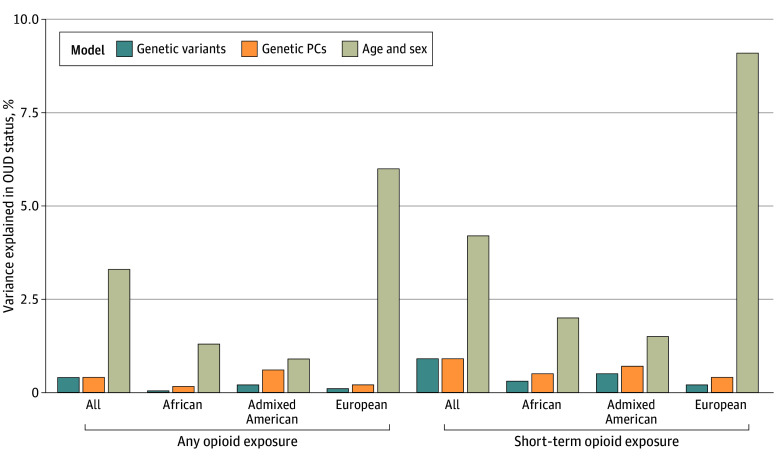
Percentage of Variance in Opioid Use Disorder (OUD) Case-Control Status Explained by Combined Single Nucleotide Variant Regression Models PCs indicates principal components.

In logistic regressions, the 15 SNVs collectively accounted for 0.40% of the variance in OUD status (Figure 1), with an AUROC of 0.54. In comparison, age and sex alone accounted for 3.27% of the variance and improved the model AUROC to 0.66. Including PCs as covariates reduced the number of SNV associations from 11 to 5. In analyses conducted within GIA groups, 7 SNVs were associated with OUD in the European group, 2 in the African group, and 1 in the admixed American group. The variance collectively accounted for by the SNVs ranged from 0.04% (African) to 0.16% (admixed American) in models within GIA groups, and AUROC ranged from 0.51 (African) to 0.53 (admixed American). eTables 3 to 6 in Supplement 1 provide full results. Compared with a model with covariates, the addition of the 15 SNVs significantly improved model fit only in individuals in the European group (χ^2^_15_ = 99.38; *P* = 1.71 × 10^−14^) and not those in the African (χ^2^_15_ = 10.02; *P* = .82) or admixed American (χ^2^_15_ = 21.50; *P* = .12) groups.

**Figure 2.**
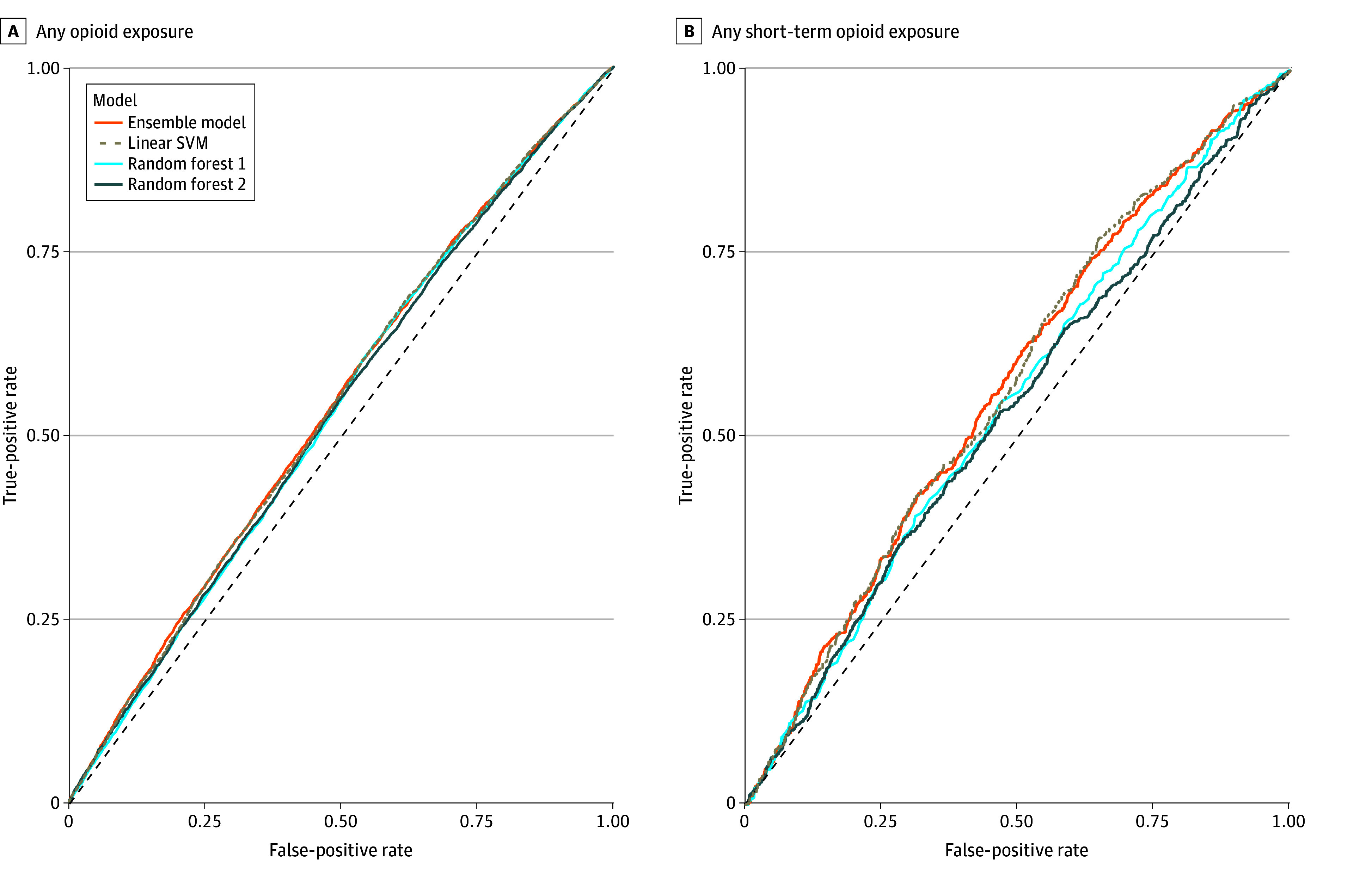
Area Under the Receiver Operating Characteristic Curves Estimating Opioid Use Disorder Case-Control Status Short-term opioid exposure indicates 4 to 30 days. Linear SVM indicates linear support vector machine model. The diagonal line represents a classifier model that predicts at chance levels.

The accuracy of the ensemble ML model in identifying OUD risk in the combined GIA sample (Figure 2) was slightly greater than random guessing (52.83%; 95% CI, 52.07%-53.59%). In test data, the model correctly identified 50.72% of OUD cases (sensitivity) and 54.95% of controls (specificity). Of the model’s identified cases, 52.96% were true cases (positive predictive value), and of the identified controls, 52.72% were true controls (negative predictive value). The DOR was 1.25 (95% CI, 1.18-1.33), suggesting that an identified case was 1.25 times as likely to have OUD than an identified control. In analyses within GIA groups that accounted for local variations in genetic similarity, accuracy did not exceed random guessing (European: 50.65% [95% CI, 49.67%-51.62%]; African: 50.53% [95% CI, 49.09%-51.96%]; admixed American: 49.69% [95% CI, 47.21%-52.16%]), and the DOR decreased to a range of 0.99 (95% CI, 0.80-1.19) for admixed American to 1.03 (95% CI, 0.97-1.14) for European. Similar results were obtained for the subsample with short-term opioid exposure (eTables 7 and 8 in Supplement 2).

**Figure 3.**
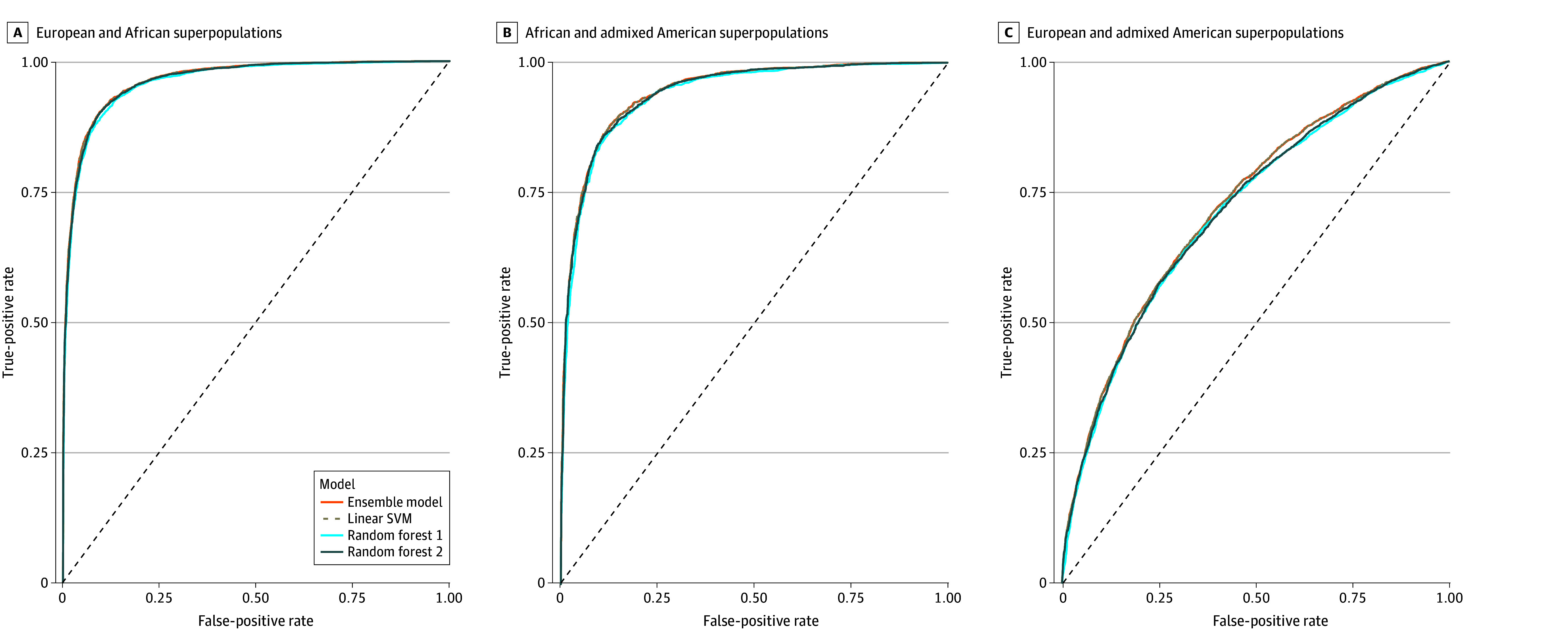
Area Under the Receiver Operating Characteristic Curves of Models Predicting Genetically Inferred Ancestry From 15 Candidate Single Nucleotide Polymorphisms Genetically inferred ancestry was based on genetic similarity to global superpopulations defined by the 1000 Genomes Project (European, African, and Admixed American). Linear SVM indicates linear support vector machine model. The diagonal line represents a classifier model that predicts at chance levels.

Confounded models assessing the ability of the SNVs to differentiate groups based on genetic similarity performed better than models identifying OUD status (Figure 3 and eTable 9 in Supplement 1). Models distinguishing individuals who were genetically similar to European from African superpopulations had an accuracy of 90.22% (95% CI, 89.63%-90.79%) and those distinguishing African from admixed American had an accuracy of 87.53% (95% CI, 86.56%-88.46%). The model was less accurate at distinguishing European from admixed American (66.07%; 95% CI, 65.15%-66.99%), likely due to the higher prevalence of genetic similarity between European and admixed American individuals.^26^

In the combined GIA models, age and sex alone yielded more accurate estimations of OUD risk (59.49%; 95% CI, 58.82%-60.16%) than the 15 SNVs. The sensitivity and specificity of the model were 0.51 and 0.68, respectively. The accuracy of models within GIA groups was comparable for individuals who were genetically similar to African (57.92%; 95% CI, 56.50%-59.33%) and admixed American (57.78%; 95% CI, 55.32%-60.21%) superpopulations and better in individuals genetically similar to the European superpopulation (63.50%; 95% CI, 62.55%-64.43%). Full results are in eTables 10 and 11 in Supplement 2.

As shown in eTables 1, 2, 7, 8, 10, and 11 in Supplement 2, the use of a stringent OUD diagnosis, where individuals with only 1 outpatient OUD diagnosis code were removed, yielded findings similar to those from the primary analyses (accuracy, 52.61% [95% CI, 51.72%-53.48%]; DOR, 1.23 [95% CI, 1.15-1.32]).

## Discussion

In this case-control study of a diverse sample of over 450 000 opioid-exposed individuals (including 33 669 individuals with OUD), we found no evidence to support the clinical utility of the 15 candidate SNVs purported to predict OUD risk. Collectively, the SNVs accounted for 0.40% of the variance in OUD risk, consistent with small individual effects of common genetic variants on complex traits.^27,28^ In an independent testing subsample, we observed high rates of false-positive and false-negative findings, with approximately 47 of 100 predicted cases or controls being incorrectly classified. False-positive findings can contribute to stigma, cause patients undue concern, and bias health care decisions.^29^ False-negative findings could give patients and prescribers a false sense of security regarding opioid use and lead to inadequate treatment plans. Notably, clinicians could better predict OUD risk using an individual’s age and sex than the 15 genetic variants. In summary, although the test approved by the FDA is intended to complement standard clinical assessment, its use is unlikely to confer additional benefits and may instead give clinicians and patients false and potentially harmful information.

We found consistent evidence that when variations in genetic similarity were not considered, it resulted in biased findings. In single-SNV logistic regressions, 10 of 13 associations (77%) with OUD risk were no longer associations when measures of global genetic similarity were covaried, with half of these reversing direction. While these SNVs were statistically null in our analysis, the FDA-approved algorithm interprets each SNV’s effect direction as meaningful for classifying OUD risk status. Thus, these direction changes are consequential to patients. The ML models also highlighted the SNVs’ ability to differentiate individuals based on their genetic similarity with high accuracy, implying ancestral confounding in unadjusted models.^14^ Notably, the FDA-approved algorithm was developed in a sample with imbalanced OUD case representation across racial and ethnic groups (genetic ancestry was not assessed).^16^ This imbalance amplifies the risk of population stratification bias, as the algorithm may learn to distinguish cases and controls based on allelic frequency patterns associated with population groups rather than true risk for OUD.

Although the AvertD test uses a proprietary algorithm, the issues identified herein suggest that the manufacturer has a fundamental misunderstanding of genetic principles, particularly the impact of differences in population structure and allele frequency. Genetics researchers have argued against the use of candidate genes to predict OUD and other psychiatric traits.^30^ Most recently, 153 genetics experts indicated concerns about use of the AvertD test in clinical settings.^31^ Whereas most SNVs in the algorithm are not associated with OUD, evidenced by both our results and multiple genome-wide association studies that failed to identify 14 of the 15 SNVs,^5,32,33,34,35^ there is limited capacity for any ML model to extract meaningful predictive value from them. Failure to account for population stratification risks embedding bias in clinical application and exacerbating health inequities.^36^

### Limitations

Several limitations of this study should be considered. First, models were evaluated using electronic health record diagnosis codes, whose assignment is susceptible to bias.^37^ However, comparable results were obtained in a sample with diagnoses assigned using structured interviews.^14^ Second, the MVP sample is predominantly male, although given its size, the analyses included over 40 000 women (over 2500 with OUD), which far exceeds the full sample on which the approved algorithm was trained and tested (1762 women and 653 cases).^16^ The MVP sample also has higher rates of OUD and pain and is older than the general population.^38^ We encourage efforts to evaluate the 15 genetic variants in additional datasets. Third, we used GIA group as a population descriptor. Despite being an improvement over previous descriptors,^39^ GIA descriptors do not fully align with recent guidance.^22^ Finally, MVP uses array genotyping, which is less accurate than that involving mass spectrometry,^40^ and imputation was required for about half of the SNVs. Although this may have reduced genotyping accuracy, it would not be expected to impact results in such a large sample.

## Conclusions

Findings of this case-control study suggest that candidate genetic variants from the approved genetic risk algorithm do not meet standards of reasonable clinical efficacy in assessing risk of opioid use disorder. Because genetic risk models in psychiatry will continue to emerge and could prove clinically useful, it is crucial that researchers and regulatory agencies adopt rigorous standards for developing and evaluating them prior to their application in clinical settings. When regulatory agencies evaluate genetic risk algorithms that use advanced statistical methods (eg, ML), it is imperative to heed the guidance of scientific advisors and independently validate the findings. By applying rigorous standards to reduce sources of bias, the potential benefits of genetic risk models can be maximized while protecting patient safety and well-being.

## Notes

Supplement 1. **eFigure 1.** Alternate Allele Frequency Across Inferred Ancestry Groups in the MVP Participants **eFigure 2.** Scatterplot of the First 2 Ancestry Principal Components Among Individuals Exposed to Opioids

Supplement 2. **eTable 1.** Full Results of Single-Association SNV Analysis in the Sample With Any Opioid Exposure **eTable 2.** Full Results of a Single-Association SNV Analysis in the Sample with Short-Term Opioid Exposure **eTable 3.** Results of Combined SNV Regression Analyses Predicting OUD Status **eTable 4.** Results of Combined SNV Regression Analyses Predicting Stringent OUD Status **eTable 5.** Results of Combined SNV Regression Analyses Predicting OUD Status Among Individuals With Short-Term Opioid Exposure **eTable 6.** Results of Combined SNV Regression Analyses Predicting Stringent OUD Status Among Individuals With Short-Term Opioid Exposure **eTable 7.** Confusion Matrices for Ensemble Machine Learning Models **eTable 8.** Confusion Matrices for Ensemble Machine Learning Models Among Individuals With Short-Term Opioid Exposure **eTable 9.** Confusion Matrices for Confounded Ensemble Machine Learning Models **eTable 10.** Confusion Matrices for Ensemble Machine Learning Models Using Demographics Only to Predict Opioid Use Disorder Status **eTable 11.** Confusion Matrices for Ensemble Machine Learning Models Using Demographics Only to Predict Opioid Use Disorder Status Among Individuals With Short-Term Opioid Exposure

Supplement 3. **Nonauthor Collaborators.** VA Million Veteran Program

Supplement 4.
Data Sharing Statement

## References

[1] Substance Abuse and Mental Health Services Administration. Key substance use and mental health indicators in the United States: results from the 2022 National Survey on Drug Use and Health. November 13, 2023. Accessed May 1, 2024. https://www.samhsa.gov/data/report/2022-nsduh-annual-national-report

[2] Dayer LE, Painter JT, McCain K, King J, Cullen J, Foster HR. A recent history of opioid use in the US: three decades of change. Subst Use Misuse. 2019;54(2):331-339. doi:10.1080/10826084.2018.1517175 30572776

[3] Tanz LJ, Gladden RM, Dinwiddie AT, . Routes of drug use among drug overdose deaths—United States, 2020-2022. MMWR Morb Mortal Wkly Rep. 2024;73(6):124-130. doi:10.15585/mmwr.mm7306a2 38358969 PMC10899081

[4] Ahmad F, Cisewski J, Rossen L, Sutton P. Provisional drug overdose death counts. Updated August 14, 2024. Accessed November 23, 2024. https://www.cdc.gov/nchs/nvss/vsrr/drug-overdose-data.htm

[5] Kember RL, Vickers-Smith R, Xu H, ; Million Veteran Program. Cross-ancestry meta-analysis of opioid use disorder uncovers novel loci with predominant effects in brain regions associated with addiction. Nat Neurosci. 2022;25(10):1279-1287. doi:10.1038/s41593-022-01160-z 36171425 PMC9682545

[6] Na PJ, Deak JD, Kranzler HR, Pietrzak RH, Gelernter J. Genetic and non-genetic predictors of risk for opioid dependence. Psychol Med. 2024;54(8):1779-1786. doi:10.1017/S0033291723003732 38317430 PMC11132928

[7] Moran M, Blum K, Ponce JV, . High Genetic Addiction Risk Score (GARS) in chronically prescribed severe chronic opioid probands attending multi-pain clinics: an open clinical pilot trial. Mol Neurobiol. 2021;58(7):3335-3346. doi:10.1007/s12035-021-02312-1 33683627 PMC8257535

[8] Donaldson K, Demers L, Taylor K, Lopez J, Chang S. Multi-variant genetic panel for genetic risk of opioid addiction. Ann Clin Lab Sci. 2017;47(4):452-456. 28801372

[9] Donaldson K, Cardamone D, Genovese M, Garbely J, Demers L. Clinical performance of a gene-based machine learning classifier in assessing risk of developing OUD in subjects taking oral opioids: a prospective observational study. Ann Clin Lab Sci. 2021;51(4):451-460. 34452883

[10] Gratten J, Wray NR, Keller MC, Visscher PM. Large-scale genomics unveils the genetic architecture of psychiatric disorders. Nat Neurosci. 2014;17(6):782-790. doi:10.1038/nn.3708 24866044 PMC4112149

[11] Crist RC, Reiner BC, Berrettini WH. A review of opioid addiction genetics. Curr Opin Psychol. 2019;27:31-35. doi:10.1016/j.copsyc.2018.07.014 30118972 PMC6368898

[12] Johnson EC, Border R, Melroy-Greif WE, de Leeuw CA, Ehringer MA, Keller MC. No evidence that schizophrenia candidate genes are more associated with schizophrenia than noncandidate genes. Biol Psychiatry. 2017;82(10):702-708. doi:10.1016/j.biopsych.2017.06.033 28823710 PMC5643230

[13] Border R, Johnson EC, Evans LM, . No support for historical candidate gene or candidate gene-by-interaction hypotheses for major depression across multiple large samples. Am J Psychiatry. 2019;176(5):376-387. doi:10.1176/appi.ajp.2018.18070881 30845820 PMC6548317

[14] Hatoum AS, Wendt FR, Galimberti M, . Ancestry may confound genetic machine learning: candidate-gene prediction of opioid use disorder as an example. Drug Alcohol Depend. 2021;229(Pt B):109115. doi:10.1016/j.drugalcdep.2021.109115 34710714 PMC9358969

[15] FDA approves first test to help identify elevated risk of developing opioid use disorder. December 19, 2023. Accessed May 1, 2024. https://www.fda.gov/medical-devices/medical-devices-news-and-events/fda-approves-first-test-help-identify-elevated-risk-developing-opioid-use-disorder

[16] AvertD package insert: instructions for use. Updated December 15, 2023. Accessed May 1, 2024. https://fda.report/media/162381/CCCTDP-102022-SOLVD-AvertD-PackageInsert.pdf

[17] Auton A, Brooks LD, Durbin RM, ; 1000 Genomes Project Consortium. A global reference for human genetic variation. Nature. 2015;526(7571):68-74. doi:10.1038/nature15393 26432245 PMC4750478

[18] Hunter-Zinck H, Shi Y, Li M, ; VA Million Veteran Program. Genotyping array design and data quality control in the Million Veteran Program. Am J Hum Genet. 2020;106(4):535-548. doi:10.1016/j.ajhg.2020.03.004 32243820 PMC7118558

[19] Delaneau O, Zagury JF, Robinson MR, Marchini JL, Dermitzakis ET. Accurate, scalable and integrative haplotype estimation. Nat Commun. 2019;10(1):5436. doi:10.1038/s41467-019-13225-y 31780650 PMC6882857

[20] Das S, Forer L, Schönherr S, . Next-generation genotype imputation service and methods. Nat Genet. 2016;48(10):1284-1287. doi:10.1038/ng.3656 27571263 PMC5157836

[21] Verma A, Huffman JE, Rodriguez A, . Diversity and scale: Genetic architecture of 2068 traits in the VA Million Veteran Program. Science. 2024;385(6706):eadj1182. doi:10.1126/science.adj1182 39024449 PMC12857194

[22] National Academies of Sciences, Engineering and Medicine. Using Population Descriptors in Genetics and Genomics Research: A New Framework for an Evolving Field. The National Academies Press; 2023:240. 36989389

[23] Chang CC, Chow CC, Tellier LC, Vattikuti S, Purcell SM, Lee JJ. Second-generation PLINK: rising to the challenge of larger and richer datasets. Gigascience. 2015;4:7. doi:10.1186/s13742-015-0047-8 25722852 PMC4342193

[24] Kuhn M. Building predictive models in R using the caret package. J Stat Softw. 2008;28(5):1-26.

[25] e1071: Misc functions of the Department of Statistics, Probability Theory Group. Version 1.7-16. September 16, 2024. Accessed November 23, 2024. https://cran.r-project.org/web/packages/e1071/index.html

[26] Wang LJ, Zhang CW, Su SC, . An ancestry informative marker panel design for individual ancestry estimation of Hispanic population using whole exome sequencing data. BMC Genomics. 2019;20(12)(suppl 12):1007. doi:10.1186/s12864-019-6333-6 31888480 PMC6936141

[27] O’Connor LJ. The distribution of common-variant effect sizes. Nat Genet. 2021;53(8):1243-1249. doi:10.1038/s41588-021-00901-3 34326547

[28] O’Connor LJ, Schoech AP, Hormozdiari F, Gazal S, Patterson N, Price AL. Extreme polygenicity of complex traits is explained by negative selection. Am J Hum Genet. 2019;105(3):456-476. doi:10.1016/j.ajhg.2019.07.003 31402091 PMC6732528

[29] Haslam N, Kvaale EP. Biogenetic explanations of mental disorder: the mixed-blessings model. Curr Dir Psychol Sci. 2015;24(5):399-404. doi:10.1177/0963721415588082

[30] Baum ML, Widge AS, Carpenter LL, McDonald WM, Cohen BM, Nemeroff CB. Pharmacogenomic clinical support tools for the treatment of depression. Am J Psychiatry. 2024;181(7):591-607. 10.1176/appi.ajp.20230657 38685859

[31] Hatoum AS, Davis CN, Kember RL, ; Ethics, Position, and Public Policy Committee of the International Society of Psychiatric Genetics, Board of Directors of the International Society of Psychiatric Genetics. Concerns about genetic risk testing for opioid use disorder. Lancet Psychiatry. Published online October 11, 2024. doi:10.1016/S2215-0366(24)00310-9 39413801 PMC12153393

[32] Deak JD, Zhou H, Galimberti M, . Genome-wide association study in individuals of European and African ancestry and multi-trait analysis of opioid use disorder identifies 19 independent genome-wide significant risk loci. Mol Psychiatry. 2022;27(10):3970-3979. doi:10.1038/s41380-022-01709-1 35879402 PMC9718667

[33] Sanchez-Roige S, Fontanillas P, Jennings MV, ; 23andMe Research Team. Genome-wide association study of problematic opioid prescription use in 132,113 23andMe research participants of European ancestry. Mol Psychiatry. 2021;26(11):6209-6217. doi:10.1038/s41380-021-01335-3 34728798 PMC8562028

[34] Gaddis N, Mathur R, Marks J, . Multi-trait genome-wide association study of opioid addiction: *OPRM1* and beyond. Sci Rep. 2022;12(1):16873. doi:10.1038/s41598-022-21003-y 36207451 PMC9546890

[35] Zhou H, Rentsch CT, Cheng Z, ; Veterans Affairs Million Veteran Program. Association of *OPRM1* functional coding variant with opioid use disorder: a genome-wide association study. JAMA Psychiatry. 2020;77(10):1072-1080. doi:10.1001/jamapsychiatry.2020.1206 32492095 PMC7270886

[36] Chung RYN, Freedman B. Health inequalities in AI machine learning. In: Sung JJY, Stewart C, eds. Artificial Intelligence in Medicine. Academic Press; 2024:119-130. doi:10.1016/B978-0-323-95068-8.00009-1

[37] Vickers-Smith R, Justice AC, Becker WC, . Racial and ethnic bias in the diagnosis of alcohol use disorder in veterans. Am J Psychiatry. 2023;180(6):426-436. doi:10.1176/appi.ajp.21111097 37132202 PMC10238581

[38] US Census Bureau. America is getting older. June 22, 2023. Accessed May 1, 2024. https://www.census.gov/newsroom/press-releases/2023/population-estimates-characteristics.html

[39] Fang H, Hui Q, Lynch J, ; VA Million Veteran Program. Harmonizing genetic ancestry and self-identified race/ethnicity in genome-wide association studies. Am J Hum Genet. 2019;105(4):763-772. doi:10.1016/j.ajhg.2019.08.012 31564439 PMC6817526

[40] Blondal T, Waage BG, Smarason SV, . A novel MALDI-TOF based methodology for genotyping single nucleotide polymorphisms. Nucleic Acids Res. 2003;31(24):e155-e155. doi:10.1093/nar/gng156 14654708 PMC291883

