# Supplementary material for: Utility of Candidate Genes From an Algorithm Designed to Predict Genetic Risk for Opioid Use Disorder
Source: JAMA Netw Open. 2025 Jan 9;8(1):e2453913. doi: 10.1001/jamanetworkopen.2024.53913 (PMC11718552; doi:10.1001/jamanetworkopen.2024.53913)
Supplement: Supplement 1. — eFigure 1. Alternate Allele Frequency Across Inferred Ancestry Groups in the MVP Participants eFigure 2. Scatterplot of the First 2 Ancestry Principal Components Among Individuals Exposed to Opioids [file jamanetwopen-e2453913-s001.pdf]

Supplementary Online Content

Davis CN, Jinwala Z, Hatoum AS, et al. Utility of candidate genes from an algorithm designed to predict genetic risk for opioid use disorder. *JAMA Netw Open*. 2025;8(1):e2453913. doi:10.1001/jamanetworkopen.2024.53913

eFigure 1. Alternate Allele Frequency Across Inferred Ancestry Groups in the MVP Participants

eFigure 2. Scatterplot of the First 2 Ancestry Principal Components Among Individuals Exposed to Opioids

This supplementary material has been provided by the authors to give readers additional information about their work.

© 2025 Davis CN et al. *JAMA Network Open*.

eFigure 1. Alternate Allele Frequency Across Inferred Ancestry Groups in the MVP Participants

Supplementary Figure 1. Alternate allele frequency across inferred ancestry groups in the Million Veteran Program participants.

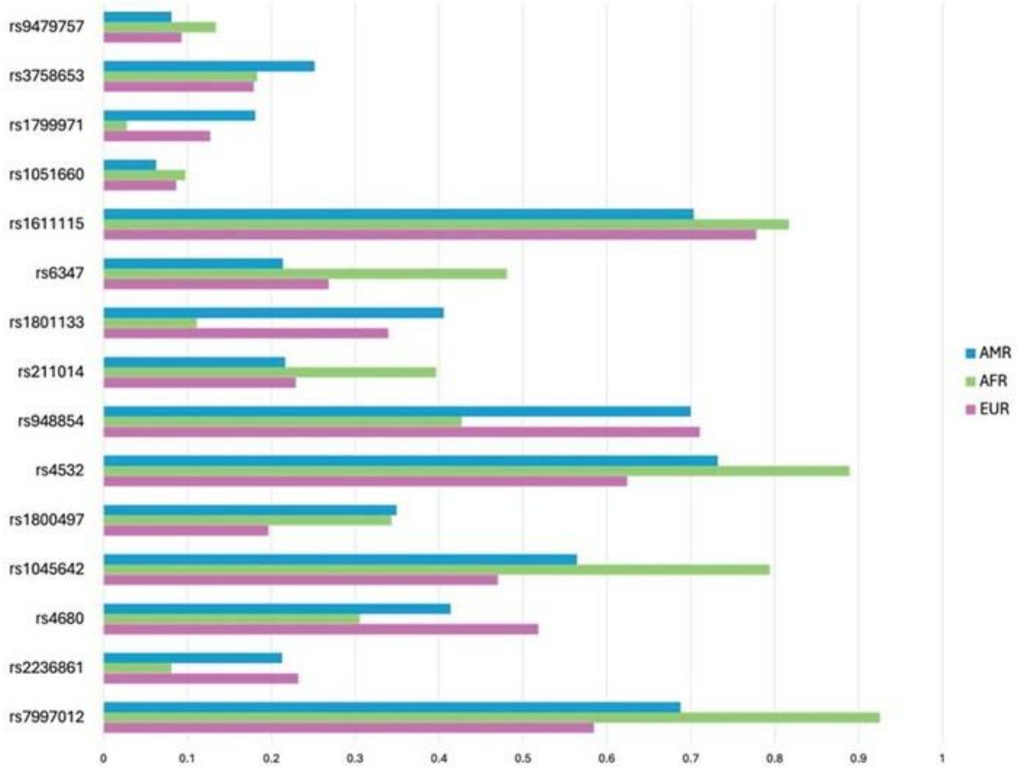

Inferred ancestry was assigned based on genetic similarity to superpopulations defined by the 1000 Genomes Project. AMR = admixed American, AFR = African, EUR = European.

© 2025 Davis CN et al. *JAMA Network Open*.

eFigure 2. Scatterplot of the First 2 Ancestry Principal Components Among Individuals Exposed to Opioids

Supplementary Figure 2. Scatter plot of the first two ancestry principal components among opioid exposed individuals.

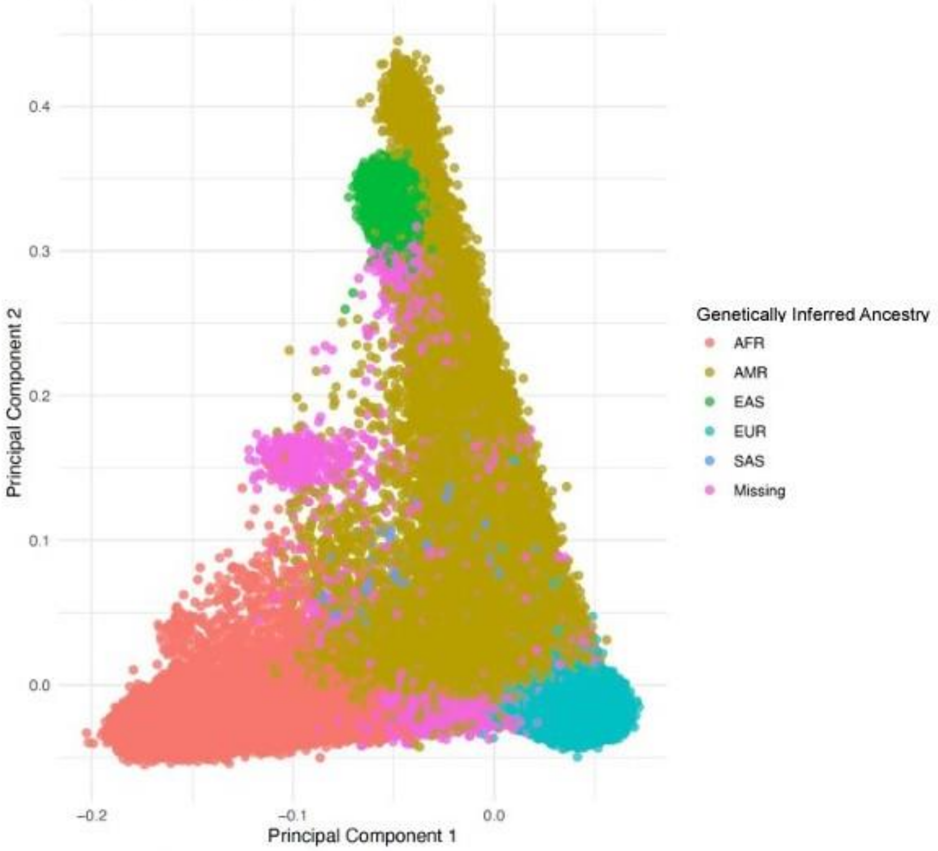

AFR = individuals genetically similar to the African superpopulation, AMR = individuals genetically similar to the admixed American superpopulation, EAS = individuals genetically similar to the East Asian superpopulation, EUR = individuals genetically similar to the European superpopulation, SAS = individuals genetically similar to the South Asian superpopulation. Individuals missing on genetically inferred ancestry were unable to be classified with a predicted probability over 50% to any given cluster based on their genetic similarity.

© 2025 Davis CN et al. *JAMA Network Open*.
