# Supplementary material for: Utility of Candidate Genes From an Algorithm Designed to Predict Genetic Risk for Opioid Use Disorder
Source: JAMA Netw Open. 2025 Jan 9;8(1):e2453913. doi: 10.1001/jamanetworkopen.2024.53913 (PMC11718552; doi:10.1001/jamanetworkopen.2024.53913)
Supplement: Supplement 4. — Data Sharing Statement [file jamanetwopen-e2453913-s004.pdf]

Data Sharing Statement

Davis. Utility of Candidate Genes From an Algorithm Designed to Predict Genetic Risk for Opioid Use Disorder. *JAMA Netw Open*. Published January 09, 2025. doi:10.1001/jamanetworkopen.2024.53913

Data
Data available: Yes
Data types: Deidentified participant data
How to access data: Database of Genotypes and Phenotypes (dbGaP), accession phs001672.
When available: With publication

Supporting Documents
Document types: None

Additional Information
Who can access the data: Researchers approved by dbGaP to access data
Types of analyses: any purpose
Mechanisms of data availability: With approval of dbGaP
Any additional restrictions: none
